# A review of post-GWAS studies in schizophrenia

**DOI:** 10.1038/s41398-025-03656-1

**Published:** 2025-10-31

**Authors:** Shahin Maserrat, Murray J. Cairns

**Affiliations:** 1 University of Newcastle, Callaghan, Australia; 2 Precision Medicine Research Program, Hunter Medical Research Institute, Newcastle, Australia

**Keywords:** Schizophrenia, Personalized medicine

## Abstract

Advances in genotyping and DNA sequencing have led to the discovery of many schizophrenia-associated loci and new and unexpected pathophysiological mechanisms. Despite this tremendous progress, only a small proportion of the Schizophrenia risk can be linked to the biological or physiological mechanisms. This has led to the development of many post-GWAS methods to further explore the mechanisms behind phenotypes and diseases. In this review we will give an overview of the current computational and experimental approaches that can convert the GWAS results into insights into the underlying causal architecture of Schizophrenia.

## Introduction

Schizophrenia (SZ) is a severely debilitating psychotic disorder with an approximate prevalence of 0.7% [1]. The disorder manifests itself through different symptom categories including, 1) Positive symptoms, including auditory and visual hallucinations and delusion or false beliefs that cannot be altered by rational argument; 2) Negative symptoms such as social withdrawal, apathy and lack of affect; 3) Cognitive symptoms such as executive dysfunction, attentional deficits and working memory dysfunction [2]. Despite several compelling neurobiological hypotheses for disease pathogenesis, including, dopaminergic, GABAergic, glutamatergic, cholinergic and serotonergic dysfunction, which emerged from neuropsychopharmacology [3–10], the exact causes that contribute to the risk of developing SZ remain unclear and highly heterogeneous. One thing we know for certain from twin studies is that the heritability is very high (77%), indicating the key role of genetic factors in the disease etiology [11]. While there was early hope that highly penetrant variants with Mendelian patterns of disease transmission may explain a substantial proportion of the phenotypic variance, these were found to be quite rare and cannot account for most individuals who present with the disorder. Research has shown that a significant proportion of the heritability can be explained by common variants with small effects, high heterogeneity and environmental exposures. For conditions like Crohn’s disease, type 1 diabetes, multiple sclerosis (MS), bipolar disorder and early onset myocardial infarction (MI) the proportion of phenotypic variance explained by common variants is 25–56%. And the proportion of heritability due to these common variants is 41–68% with an average of 60%. So common variants account for at least half of the heritability in many diseases, so they are a major determinant in complex traits [12, 13].

**Fig. 1:**
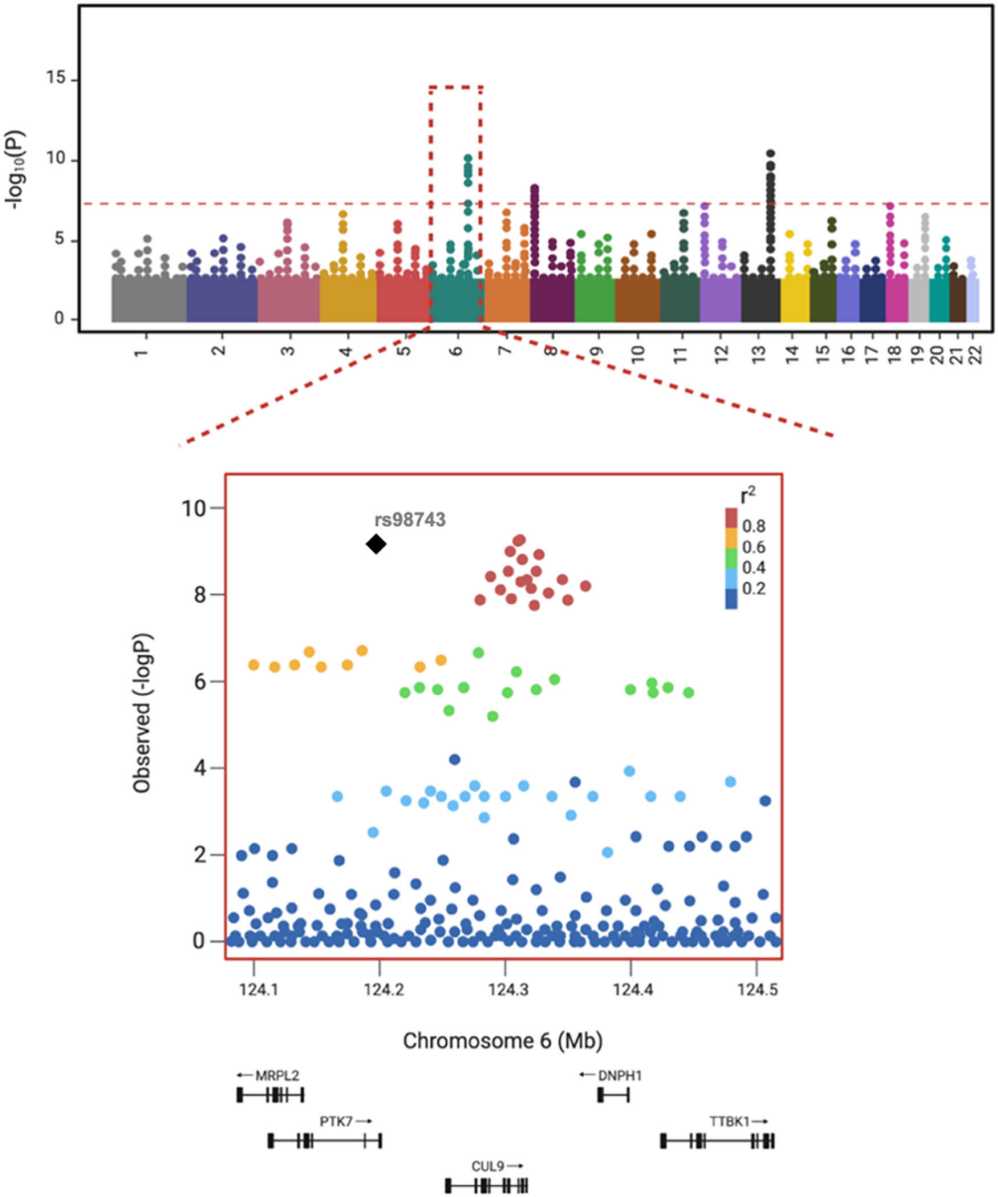
Schematic Genome-wide association studies results. A Manhattan plot visualizes the association between genetic variants and a phenotype of interest. The plot displays the -log10 (p-value) for each genetic variant on the x-axis, with the y-axis representing the chromosome position of the variant. The top panel shows the results of the association analysis at a genome-wide level, meaning all genetic variants across the entire genome are included in the analysis. The significant threshold line represents the threshold for statistical significance, which is usually set at a p-value of 5×10^-8^ in genome-wide association studies. The bottom panel displays the results within a specific locus, which is a region of the genome that is of interest. This panel allows for a closer examination of the association between genetic variants and the phenotype of interest within a specific region of the genome. The black diamond represents the index SNP. Other SNPs are colored based on their r² values with the index SNP: red (high LD) and dark blue (very low LD).

The last 15 years has seen very tangible progress in the discovery of common variants associated with SZ using GWAS. In this approach genetic variation is mapped across the genome in large numbers of individuals to determine the difference in allele frequency related to complex diseases and other traits [14]. SNP-array technology has been instrumental in enabling cost effective genome-wide analysis of large numbers of individuals to determine which loci (represented by common single nucleotide polymorphisms (SNPs) are associated a binary phenotype or differences with respect to a continuous trait. This is visualised in Manhattan plots which presents the -log10 of the p-values (Fig. 1). Since variants are correlated with each other (what is known as *linkage disequilibrium (LD)*), the specific causal variant and causal gene is often obscured, particularly when they occur in non-coding segments and affect gene regulation/expression. Over 80% of GWAS hits map to non-coding regions, while fewer, like missense variants, occur in coding regions and directly impact protein function [15].

**Fig. 2:**
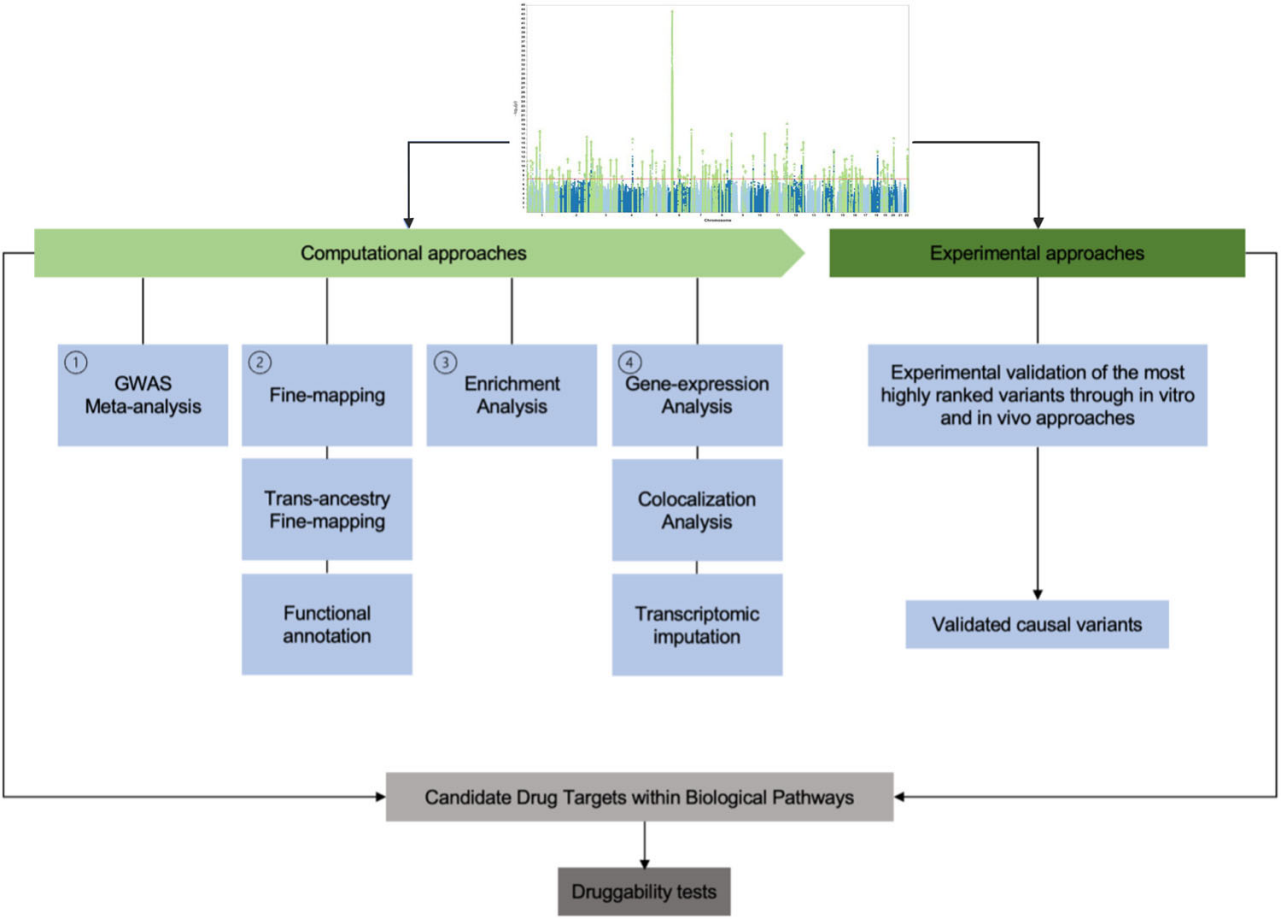
Flowchart of post-GWAS process for identifying causal variants and genes in SZ based on GWAS outcomes. (1) GWAS Meta-analysis combine multiple GWAS studies to increase statistical power and identify common variants associated with SZ. (2) Fine-mapping candidate variants involves narrowing down the list by analysing additional genomic data, such as linkage disequilibrium patterns or functional annotations. This process also includes trans-ancestry fine mapping to identify variants with different frequencies and potential population-specific effects. Additionally, leveraging functional annotation data helps prioritise variants likely to have functional implications, such as regulatory elements or chromatin states. (3) Enrichment Analysis assess enrichment of SZ-associated variants in specific biological pathways or gene sets to gain insights into underlying biological mechanisms. (4) Performing enrichment analysis, the study involves colocalization analysis to assess the overlap between GWAS signals and eQTL data, aiding in the identification of potentially causal genes. Additionally, transcriptomic imputation is employed to predict gene expression levels using genetic data, facilitating the identification of genes that are dysregulated in SZ. To validate the functional significance of top-ranked variants, a thorough experimental approach using in vitro and/or in vivo methods is essential to confirm their involvement as causal variants in the anticipated phenotype. By integrating the data from GWAS and post-GWAS analysis, it is possible to uncover potentially druggable gene and/or protein pathways, leading to new discoveries and insights, such as in the case of SZ.

The establishment of the Psychiatric Genomics Consortium (PGC) led to unprecedented global cooperation and tremendous progress in the mapping of genomic loci associated with SZ [16–18] including the latest wave 3 study yielding 287 independent significant loci. Despite these unprecedented discoveries, the clinical insight obtained from GWAS results have been controversial due to two major complicating factors. The first of these is that the SNP effects are generally small and highly heterogeneous. The second, is that most significant loci ( > 95%) localise to non-coding regions of the genome, and it is often ambiguous what the functional effects are or even what genes are involved [19]. Non-coding regions of the genome contain regulatory elements (promotors and enhancers) that can play regulatory roles for gene expression through binding transcription factors (TFs) proteins. The function of these TFs can be disrupted by these variants which result in altered promoter or enhancer activity. The major challenge in assigning causality of GWAS hits is that most common genetic variants are inherited in haplotype which make it difficult to distinguish the causal variants driving the association from the index-SNPs and other variants that are in high LD. These issues have collectively hampered the biological and pharmacological translation of GWAS data. Despite these challenges, new methodological approaches, including both statistical methods and molecular techniques, are making progress in post-GWAS analysis to identify the and associated genes to facilitate the interpretation of their biological role in SZ risk. In this review, we summarize various current computational and experimental approaches that facilitate translation of GWAS outcomes for identifying causal variants and genes in SZ and we moreover present the key results of these studies (Fig. 2).

## GWAS and Post – GWAS analysis in SZ

Whilst several GWAS of SZ have been published over the last decade, many early studies were unable to detect more than a handful of genome-wide significant loci [16, 20, 21]. Large scale international collaboration has taken place in the recent decade to enable the shift from studies, which discovered one genome-wide significant locus for SZ in 479 samples [16] to a combination of datasets up to 69,000 SZ individuals identifying 287 significant loci [18]. The first wave of this global effort coordinated by the PGC, consisted of 17,836 cases and 33,859 controls and revealed seven genome-wide significant loci [22]. Additional samples with a total size of 38,072 controls and 21,246 cases from a Swedish cohort were combined with data from this study, finding 22 significant loci, thirteen of these were novel associations [23]. The second wave data from the PGC produced what was likely the most important discovery in SZ genetics, consisting of more than 36,989 cases and 113,075 controls, which, together with a replication sample and parent offspring sample, had enabled the discovery of 108 distinct loci associated with SZ. These finding provide new insight into the biological pathways and multiple candidate genes of potential therapeutic relevance in SZ [24]. Another large scale GWAS through meta-analysis with new SZ sample from the United Kingdom (UK) (CLOZUK sample) built on this work by Pardinas et al. [25], where more than 100,000 samples implicated 145 loci associated with SZ. To date, the latest wave 3 study by PGC have mapped common variant associations at 287 distinct loci that involve 69,369 cases and 236,642 controls.

Although GWAS have identified hundreds of genetic risk factors for SZ, it is essential to consider how this knowledge can be applied to improving treatment. Post-GWAS analyses provide insight into the molecular etiology of several complex disorders and these approaches are being applied to SZ. However these methods are not without limitations [26]. A significant challenge of the post-GWAS era is understanding the genetic risk factors that cause functionally significant biological effects. For example, studies often focus on the expression levels of genes near associated variants, such as the CACNA1C gene, which has been linked to SZ and influences calcium channel activity in neurons [27, 28]. Knock-out experiments in animal models, such as mice lacking the DISC1 gene, also help elucidate gene function and its impact on SZ [29, 30].

These findings reinforce the clinical benefits that can be derived from biological insights, including methods that are effective for screening and prevention of disease based on reliable biomarkers. For instance, the identification of the complement component 4 (C4) gene variant has led to the development of potential biomarkers for SZ [31]. Post-GWAS analysis approaches can be grouped into two broad categories: the first, bioinformatic tools including: 1) single-variant tests; 2) gene-based association tests; 3) pathways based approaches [32]. The second category, is molecular biology techniques including CRISPR/Cas 9 genome editing, reporter assays and EMSAs. These approaches are discussed in detail below. For statistical approaches, a list of the most commonly used web applications and software package approaches for SZ are provided in Table 1.

**Table 1:** Summary of most common methods in post- GWAS studies for SZ.

Tools	Description	Website link	ref.
**Single- variant association approaches**			
FINEMAP	A tool for fine-mapping analyses which uses a shotgun stochastic search algorithm to effectively exploring the region with the most significant causal component.	http://www.christianbenner.com	[130]
METASOFT	Fixed, random and binary effects model based on inverse-variance-weighted effect size to detect associations under heterogeneity.	http://genetics.cs.ucla.edu/meta	[44]
Sherlock	Sherlock uses Bayesian statistical framework to match genetic gene expression signatures from eQTL with GWAS association for identifying gene associated with disease.	http://sherlock.ucsf.edu	[131]
SMR	Utilising summary-level data from GWAS and eQTL studies, to examine potential pleiotropic associations between a complex trait of interest and the level of gene expression. By analysing genetic variants and their effects on both the trait and gene expression, this approach provides insights into whether there are shared genetic influences influencing both the trait and gene expression patterns.	https://hpc.nih.gov/apps/SMR	[93]
CAVIAR	A statistical framework that assess the likelihood of each genetic variant being responsible for a particular trait or disease. It allows for the consideration of multiple potential causal variants within a specific genomic region. By estimating the probability of causality for each variant, CAVIAR provides valuable insights into the genetic architecture of complex traits and diseases.	http://genetics.cs.ucla.edu/caviar	[132]
METASOFT	Enables trans-ancestry studies through a modification of the traditional random-effects methods.	http://genetics.cs.ucla.edu/meta	[44]
**Pathway based approaches**			
MAGMA	A flexible and fast method for gene-set and gene analysis form GWAS data.	https://ctg.cncr.nl/software/magma	[133]
INRICH	Identifying enriched association signals in predefined gene sets across independent genomic intervals. It helps to detect significant associations within specific gene sets across different regions of the genome, revealing potential biological mechanisms and pathways related to a phenotype or trait.	http://atgu.mgh.harvard.edu/inrich	[134]
MAGENTA	Assessing the enrichment of genetic variants associated with a specific disease or trait within gene sets. It achieves this by analysing disease association p-values and odds ratios from genome-wide association studies (GWAS) to identify modest associations.	https://software.broadinstitute.org/mpg/magenta/	[135]
PASCAL	Enables the generation of gene scores by combining SNP p-values from GWAS meta-analysis while accounting for the influence of LD structure. By considering LD, PASCAL ensures more accurate and reliable results when assessing the genetic associations within a pathway or trait of interest.	https://www2.unil.ch/cbg/index.php?title=Pascal	[136]
GSA-SNP	GSA-SNP is JAVA-based stand-alone software, that implements the three GSA methods including: GSEA, Restandardized GSA, Z-statistic method.	http://gsa.muldas.org	[137]
**Gene-based approaches**			
FUSION	TWAS software	https://github.com/gusevlab/fusion_twas	[100]
PrediXcan	Assessing the cumulative impacts of cis-regulatory alterations on gene expression is achieved using an elastic net regression approach. As a result, PrediXcan has the potential to detect genomic regions characterized by subtle to moderate effect magnitudes, which might not attain statistical significance in association studies reliant on variants.	https://github.com/hakyimlab/PrediXcan	[101]
VEGAS	Pooling data from the complete array of SNPs situated within a gene, this method considers the influence of linkage disequilibrium (LD) through simulations based on the multivariate normal distribution.	https://genepi.qimr.edu.au/general/softwaretools.cgi	[138]
GATES	This technique is applied within a software tool named Knowledge-guided mining system for Genome-wide Genetic studies platform (KGG). It employs an expanded Simes method to appraise the statistical importance of associations at the gene level.	http://bioinfo.hku.hk/kggweb/	[139]

*SMR* summary-based mendelian randomization, *CAVIAR* causal variants identification in associated regions, *MAGMA* multi-marker analysis of genomic annotation, *INRICH* interval enrichment analysis, *MAGENTA* meta-analysis gene-set enrichment of variant associations, *PASCAL* pathway scoring algorithm, *GATES* gene-based association test using extended simes procedure, *VEGAS ve*rsatile *g*ene-based *a*ssociation *s*tudy.

## Using single-variants association approaches in post- GWAS for SZ

### GWAS Meta-analysis

Although many genetic variants are identified by single GWASs, these variants only elucidate a proportion of the phenotypic variance for SZ. Common alleles have small genetic effects, and large sample sizes are required to detect signals [33]. To overcome this issue, large consortia such as PGC, consider combing multiple GWASs into a single aggregate analysis for boosting power to detect associations of SNPs with small effects for SZ. In human genetics, GWAS meta-analysis based on summary-level data has become a popular important approach to increasing power while avoiding the logistical problems associated with sharing individual-level data. Meta-analysis is a statistical procedure used to integrate data from multiple independent studies for increasing power and reducing false-positive results [34]. In meta-analysis, there are two main models: fixed effects and random effects. Fixed effects is very powerful and popular for finding and prioritising phenotype associations with SNPs by pooling data from GWAS [35]. This model assumes the true effect of the risk allele is the same across all datasets which can increase power by pooling data under the assumption of a constant effect size. For diseases with strong, consistent genetic signals, such as monogenic disorders, fixed effects models give more precise estimates as they aggregate data under the assumption the effect size is the same across studies. This is especially true when the studies have similar sample sizes and genetic architecture or when the population is relatively homogeneous in terms of genetic or environmental diversity.

In contrast, the random effects model assumes the causal effect sizes can vary across studies. This model accounts for the variability between studies which can arise from differences in study design, sample characteristics or population diversity. For example, if the study subjects vary in health status or age the effect size may differ and random effects models can accommodate this variation. While random effects models add extra uncertainty they can be statistically conservative by identifying associations that withstand this extra variability. This approach is useful for complex traits or diseases like psychiatric disorders where genetic and environmental factors are highly heterogeneous. So random effects models are generally more suitable for these conditions as they give a broader view of genetic influences and account for variability across studies.

When discovery is the main goal fixed effects can be more powerful as they use the assumption of uniform effect sizes to give more precise estimates. But for complex diseases with a lot of variability like SZ random effects are often preferred to account for the heterogeneity and give a more detailed view of the genetic data [36, 37]. In recent years, increasingly large GWAS meta-analyses have been performed for individuals of several ethnic populations, especially in European ancestry. As of the time of this review, the PGC has reported the largest GWAS for SZ through meta- analysis [18].

### Fine-mapping candidate variants

**Fig. 3:**
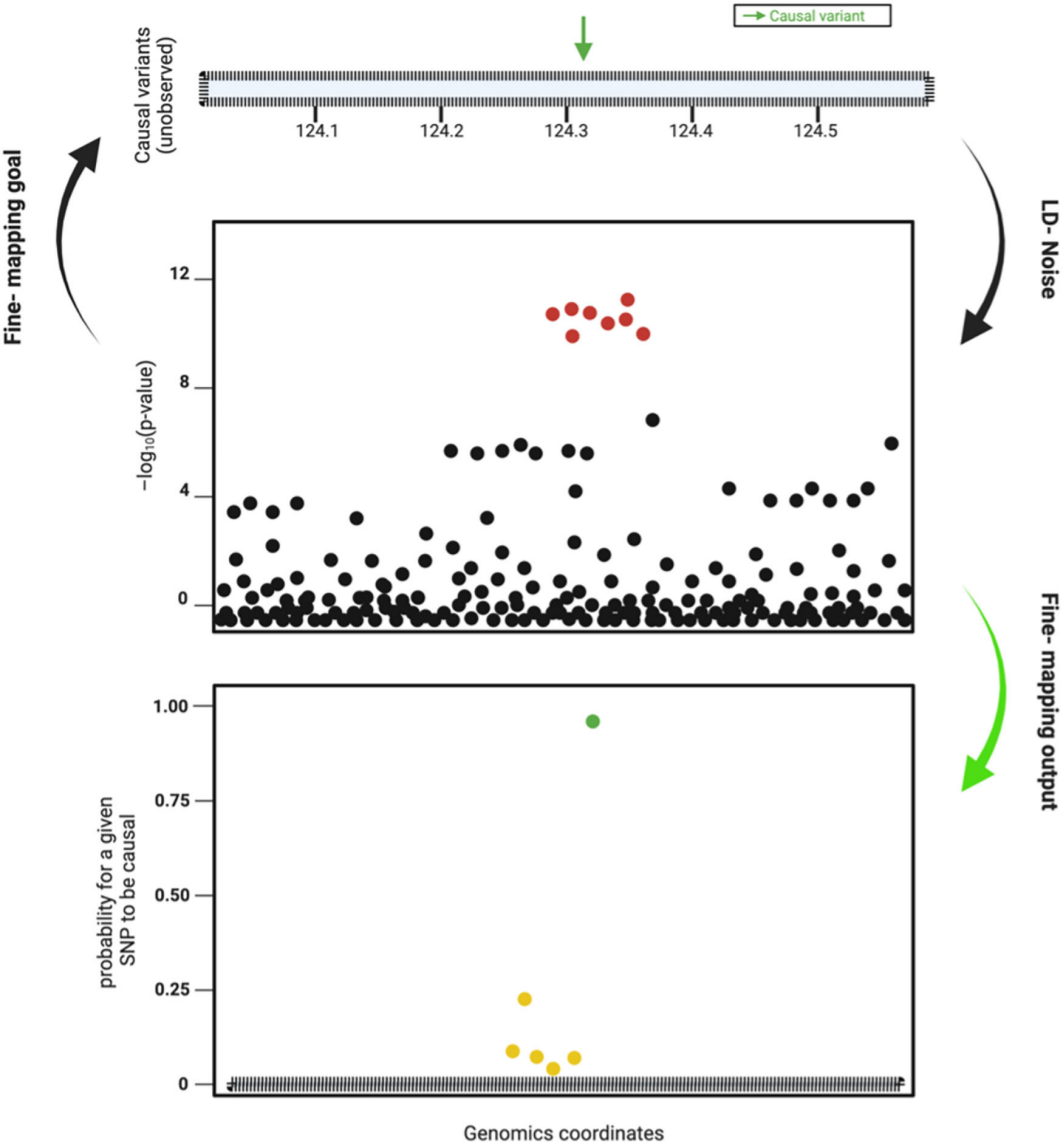
Statistical fine-mapping of genetic associations. **Top panel**. A schematic representation of the genomic region with potential causal variants. The green arrow indicates the true causal variant among many unobserved variants. **Middle panel**. A plot showing the -log_10_ (p-value) of SNPs across the genomic region. The red dots represent SNPs with strong association signals, influenced by LD with the true causal variant, resulting in multiple SNPs showing significant p-values due to LD noise. **Bottom panel**. A plot showing the probability of each SNP being causal based on fine-mapping analysis. The green dot indicates the SNP with the highest probability of being causal, correctly identifying the true causal variant. Yellow dots represent SNPs with moderate probabilities.

While the causal SNP in an LD block of a GWAS association signal is difficult to resolve, statistical fine-mapping provides the means to narrow down the candidates prior to functional validation. Fine-mapping essentially ranks variants according to their marginal association strength (i.e. prioritisation of p-values) [38] (Fig. 3). Although relatively straight forward for single causal variants, it can be challenging to optimise if regions contain several causal variants.

The standard method for identifying causal variants has been conditional analysis, which utilises a stepwise greedy forward selection approach. This approach begins by selecting the variant with the lowest P-value from the GWAS and then proceeds to choose the variant with the lowest conditional P-value in an iterative manner. The process continues until either no further variants reach the genome-wide significance threshold or a predetermined number of iterations have been conducted [39]. There are several algorithms for fine-mapping, each utilising different genetic association models and considering the variety of functional annotations (as reviewed by Schaid et al. [40]). For instance, in a Bayesian approach, each set of SNPs in a subregion is assigned a prior probability of being the ‘true’ model, and posterior probabilities are then used to compute posterior distributions for each sub region which are then combined. The resulting posterior distribution for the region models the probabilities for each SNP to be causal. This relies on the probability of the observed z-scores considering different potential combinations of causal variant(s) [41]. These posterior probabilities enable the formation of a credible group of SNPs, characterized as the most compact assortment of SNPs that includes the actual causal variant(s) with a specified probability. Fine mapping in the largest GWAS of SZ has prioritised 19 genes based on nonsynonymous variants and a single UTR variant in *CACNA1I (*voltage gated calcium channel subunit*)*. These variants including the non-synonymous substitution in SLC39A8 which mediates uptake of manganese and zinc, a variant in ZNF823 [18].

#### Trans-ancestry fine mapping

Using diverse ancestries in fine-mapping could be an effective method for identifying true causal variants responsible for the observed associations. Trans-ancestral meta-analyses that combine GWAS across different ancestry groups accounts for differences in LD and the varying allelic effects and frequencies observed between ancestries. Assuming a shared causal variant between ancestry groups, the surrounding variants in LD with the causal variant may differ slightly between ancestries. Statistical power for fine-mapping can be improved using multiple ancestries by increasing sample size and taking advantage of differences in LD structure between genetically heterogeneous populations [42, 43]. As the set of SNPs tagged to each causal variant will differ among populations, signals from causal variants will be strengthened, while signals from the index SNPs will be diluted by aggregating evidence of association across populations. Trans-ethnic analyses are conducted using random-effects methods as meta-analyses of summary statistics. This approach helps to identify distinct effect sizes of SNPs across diverse populations. SNP and LD heterogeneity can be caused by differences in interactions with other SNPs, differences due to lifestyle, cultural, or environmental factors and differences in study designs. One of the most popular tools for performing trans-ethnic studies, METASOFT, implements a modification of the traditional random-effects methods [44]. Trans-ethnic fine-mapping can also be accomplished with CAVIAR which estimates the posterior inclusion probabilities (PIPs) and determines credible sets through functional annotation.

Using this approach the genetic architecture of SZ in European and East Asian samples, was compared (Lam et al. in [45]). The study revealed that the common variant genetic architecture of SZ outside of the major histocompatibility complex (MHC) region is highly consistent across these two populations. However, the polygenic risk calculated for East Asians was found to be different from that of Europeans, which can be attributed to differences in allele frequencies, LD, and other factors based on ancestry [45].

#### Leveraging functional annotation data

In recent years, large research efforts such as the Encyclopedia of DNA elements (ENCODE) consortium [46] and National Institutes of Health (NIH) Roadmap Epigenomics project [47] have been underway to make available data for understanding functional noncoding elements at the epigenomic level. There is an opportunity to perform interpretation of noncoding variants by using these data sources. As mentioned earlier, a major part of complex trait associated genetic variants are located in noncoding regions which can act as cis or trans- regulatory elements in the regulation of gene expression. In general, these include the enhancer and promoter regions, TFs-binding sites, DNase I hypersensitive sites, histone modification sites and DNA methylation sites, which are involved in biological process including cleavage, transcription, and translation of genes [48, 49]. Therefore, functional annotation of GWAS signals enable us to identify functional SNPs associated with various regulatory processes [49–51]. Current functional annotation databases can be incorporated into analyses of statistical associations for improving both the GWAS detection power and fine mapping resolution underlying causal variants [52, 53]. Initial studies have demonstrated variants associated with disease are enriched in chromatin markers that delineate active regulatory regions in phenotypically relevant cell types [48, 54]. As a consequence of this pattern, several approaches have been developed to jointly integrate association and functional data and update posterior probabilities of association (PPAs) for functional enrichment analysis [55]. Analysis of comprehensive annotation of chromatin, indicated that functional SNPs are broadly involved in diverse biological processes. For example, histone modifications including H3K4me3, H3K4me1, H3K36me3 and H3K27ac were found to be enriched in SZ [56].

## Using biological pathways in post GWAS analysis of SZ

In pathway-based tests, genes annotated by GWAS have been analysed to determine a group of genes which are in similar molecular and/or biological processes underlying complex diseases. GWAS genes can be mapped into molecular pathways [57] and gene networks [58] to improve understanding of SZ biology risk loci. Moreover, we can uncover future potential drug targets by investigating relevant biological pathways. For example, pathway studies of SZ based on genomic data have converged on synaptic mechanisms presenting novel drug target opportunities [59].

### Enrichment Analysis

Enrichment analysis is an important approach for prioritising genes and variants from GWAS, among SNPs below the genome-wide significance level. While GWAS have successfully identified genome-wide significant variants for many diseases and traits, these variants only account for a minority of the trait heritability. Enrichment analysis takes a different approach by testing disease association at the level of a group of functionally related SNPs or genes, such as those belonging to the same biological pathway. This approach avoids the need to conduct analyses for every single SNP or gene and can help prioritise potentially relevant variants. Identification of genes and their corresponding biological pathways that are associated with SZ will considerably increase our knowledge of the biological mechanisms of SZ pathogenesis. It is important to note that comprehensive gene lists alone are insufficient to produce a deeper understanding of drug targets or mechanisms of disease. A variety of approaches have been proposed over the past few years to investigate significant pathways and put large SNP effect size collections generated by GWAS in a broader biological context [60, 61]. These approaches aim to increase power by combining association signals.

Gene set analysis (GSA) is a common complementary strategy in GWAS that extends the information from single genetic variations to their impact on the biological processes underlying complex diseases. GSA is valuable for pathway analysis, annotating biological features using genetic association study results. There exist various tools and methods for GSA (Table 1), with differing practical and statistical complexities. Although these tools differ, their statistical analysis structures are similar. Initially, genotype data are used to calculate associations between phenotype and each gene. The results are then reported as a data matrix containing rows corresponding to genes, including scores of true associations for each gene and indicator variables encoding the gene set. A bivariate analysis is performed on this gene-level data matrix to determine the relationship between gene-association scores and gene-set indicators.

Originally, GSA used data from gene expression analyses [62], but it has since been shown that sequence variation is also associated with biologically segmented gene sets. This includes phenotypes such as SZ [17], bipolar disorder [63, 64] and susceptibility to breast cancer [65]. Some GSA analyses have provided novel biological insights into complex disorders, potentially opening new therapeutic avenues [22, 66]. For example, recent studies showed that growth factor signalling pathways are involved in multiple cancers [67]. Pathway analysis of neuropsychiatric disorders such as bipolar disorder, SZ and depression has revealed numerous genetic variants associated with neuronal and immune pathways [68]. Gene set analysis performed using GWAS data with post-mortem brain gene expression data has reported that ion channels and calcium signaling are involved in SZ [69]. GSA tools require biological annotations from databases such as Reactome [70], Gene Ontology (GO) [71] and Kyoto Encyclopedia of Genes and Genomes (KEGG) [72] which are the most widely used functional gene database to perform functional annotations for genes in SZ. Biologists are able to interpret gene functions using all of these databases together. Pardiñas et al. [25] conducted a systematic analysis of the association of functional gene sets with SZ as part of the largest GWAS meta-analysis by using a the competitive gene set enrichment analysis approach (MAGMA). They highlighted six gene sets that are independently associated with SZ, pointing to behavioral, molecular and physiological pathways involved in pathogenesis of SZ [25]. The gene sets included the targets of the fragile X mental retardation protein (FMRP) which showed the strongest association with SZ [73]. FMRP as a neuronal RNA binding protein, interacting with mRNA polyribosomal, suggesting that it could act to inhibit target mRNA translation. These analyses also reveal gene sets that are independently associated with SZ, including genes related to neurophysiological correlates of learning and behavioral (abnormal long-term potentiation, abnormal behavior and abnormal nervous system electrophysiology) genes associated to voltage-gated calcium channel complexes [74] and genes encoding protein of 5-HT_2C_ receptor complex [75]. This discovery confirmed previous findings from rare and common variant studies that implicated calcium channel genes in SZ [24, 76]. Additionally, a comprehensive integrated pathway analysis of gene expression and GWAS data observed that the main type of pathways that are enriched in genes associated with SZ are related to cell adhesion, immune system, neurodevelopment, synaptic function, neuronal functioning and apoptosis [20, 77–79]. Furthermore, these studies reported CTCF and CACNB2 genes, involved in chromatin modulation and calcium channels, were candidate genes for SZ risk. CTCF has also been shown to regulate the expression of genes implicated in neurodevelopmental disorders such as autism [80], Spinocerebellar ataxia type 7 [81] and neurodegenerative diseases, such as Huntington’s disease [82].

## Using gene-expression based association in post-GWAS analysis

In gene-based approaches, instead of examining each marker (SNP) individually, we consider the relationship between all markers and trait within a gene. The gene-based tests compared to individual-SNP based GWAS (traditional methods) are potentially more powerful, depending on the underlying genetic basis. As an example, when more than one causal variant exists for a gene, several SNPs within that gene might exhibit low significance levels, which can sometimes resemble random noise in the initial GWAS outcomes. By consolidating signals from various SNPs within the gene into a single test-statistic and addressing LD-related issues, the gene-level test could potentially identify these effects.

### Colocalization

The functional consequences of protein coding variants is now well understood and when they occur it is relatively straight forward to predict their potential role in human health and disease. However, the protein coding sequence only represents a tiny fraction (~%1) of the genome and we now know that most disease associated genetic variation occurs in non-coding segments. Rather than altering protein structure these disease-associated non-coding variants occur in genomic regulatory regions and alter gene expression [83–85]. While it is more difficult to ascertain the role of these vasriants the development of next-generation sequencing techniques has facilitated greater functional analysis of non-coding regions to enable the comprehensive analysis of quantitative trait loci (QTL) through the relationship between DNA sequence and gene expression.

QTL refers to DNA markers on a chromosome that indicate genes associated with a quantitative trait [86]. In recent years, QTL analysis has been to identify loci linked to specific quantitative phenotypic traits caused by polygenic effects [87]. This approach has been beneficial in assessing human polygenic diseases and phenotypes, as well as intermediate phenotypes such as gene expression. In the context of SZ, these analyses help to identify loci associated with the disease, providing insights into the genetic architecture and underlying biological mechanisms. Moreover, eQTL analysis uncovers disease risk loci and associated genes that may influence pathogenesis in various diseases, including cardiovascular disease [21], diabetes [22], inflammatory bowel disease [23], epilepsy [24], SZ [25], and cancer [26]. Additionally, eQTL analysis has been utilized to assess the impact of genetic variants on cellular responses to external stimuli, highlighting its clinical utility in identifying potential therapeutic targets [27]. This expanding field of research not only deepens our understanding of complex diseases but also paves the way for developing targeted therapies and personalized medicine approaches.

Many large QTL studies have been developed as a result of rapid advancements in genome sequencing technologies and functional genomics such as “chromatin profiling”, which link phenotypic and genotypic data to explain the genetic composition of complex traits based on two types of information. QTL mapping has been used for studying the genetic determination of splicing (sQTL) and gene expression (eQTL) in transcriptional regulation, histone modification (hQTL), DNA methylation (mQTL) in epigenetic regulation and chromatin accessibility (caQTL), cell metabolism (metaQTL) in post-translational regulation, competing endogenous RNA expression (cerQTL) and RNA editing (reQTL) in post-transcriptional regulation, protein expression (pQTL) and ribosome occupancy (riboQTL) in translational regulation [88–90]. Of all these, eQTLs are the most commonly utilised, because of the power of RNA sequencing technologies. The integration of transcriptomic studies and GWAS via imputed expression has been the front-line method in recent post-GWAS studies in SZ, as it enables causal gene prioritisation and functional interpretation of GWAS loci. Several databases/resources are available for eQTL information. Several databases and resources provide valuable eQTL information. The MetaBrain study stands out as one of the largest brain eQTL resources available, offering comprehensive insights into the genetic regulation of gene expression in the brain (https://metabrain.nl/). This extensive dataset is a valuable asset for researchers conducting colocalization studies involving brain tissue. Another significant resource is the Genotype-Tissue expression project (GTEx), which is one of the most comprehensive eQTL database. GTEx was established to build a public resource for studying tissue-specific regulation and gene expression (profiled 53 tissues across nearly 1000 individuals) [91]. Another prominent resource of eQTL is the CommonMind consortium which provided transcriptomic and epigenomic data for SZ, where gene expression of dorsolateral prefrontal cortex was analysed in over 500 SZ cases and controls [92]. It is theorised that GWAS SNPs are enriched with eQTLs [93, 94]. Integrating GWAS with QTL maps can enable identification of a possible molecular mechanism for disease association. As shown by Nicolae et al. [94], concluding that, SNPs reported by GWAS have almost twice the chance to be eQTLs than arbitrary sets of SNPs by combining eQTLs from human lymphoblastoid cell lines and GWAS finding [94]. Looking for overlaps between eQTL variants and variants associated with complex traits has been effectively utilised as evidence of a common causal molecular mechanism. Three main scenarios could explain overlaps between GWAS and eQTL signals: (1) There is LD between two independent causal SNPs (Linkage), (2) Traits and gene expression are affected by a single causal SNP (causality), or (3) a single causal SNP with independent effects on gene expression and trait (pleiotropy). It is essential to distinguish between these scenarios for appropriately interpreting GWAS findings. Colocalization allows us to assess whether two genetic association signals (e.g., both GWASs and eQTLs) are consistent with shared causal variants. Hence, colocalization requires correctly identifying the causal variant in both studies. Several tools are available to facilitate colocalization analysis. One notable tool is **coloc** (https://github.com/chr1swallace/coloc), which uses Bayesian methods to estimate the likelihood that two genetic signals originate from the same causal variant [95]. Several studies have shown plausible risk genes for SZ by combined approaches to integrate eQTL data sets and genetic variation associated with GWAS signals. It has been demonstrated by several recent studies that migration and proliferation of neural progenitor cells can be regulated with genes related to SZ such as *RELN* and *DISC1* [96, 97]. The authors also showed that *CSNK2B*, *GLT8D1* and *ALMS1*, contribute to pathogenesis of SZ, by affecting proliferation and inhibiting the differentiation abilities of neural stem cells. They also alter morphology and synaptic transmission of neurons, providing more evidence in support of the neurodevelopmental hypothesis of SZ. Furthermore, several genetic factors (such as MMP16, TCFA, CNTNA, PSMA, GATADTA and GPMNA) play a role in the transmission of signals between neurons and the development of the brain, which adds to the theory that SZ is linked to neurodevelopment. Worth mentioning is TCFAs involvement in communication between nerve cells the ability of synapses to change in response to activity (synaptic plasticity) and the general upkeep of synapse integrity and functionality. Key aspects, for cognitive abilities and brain functions [98, 99].

### Transcriptomic imputation

Testing direct associations between gene expression and traits is another avenue to gain insight of complex traits biology. Unfortunately, the lack of specimen availability and costs have hampered the direct measurement of gene expression in complex trait studies. However, when the effect of genotype on gene expression are known, a Transcriptome Wide Association Study (TWAS) can be conducted using imputed changes in gene expression associated with the trait. Hence, researchers have developed methods to allow indirect measurement of gene expression and discover genes whose level of expression is significantly correlated with complex traits. Fusion [100] and PrediXscan [101] are examples of predictive tools to perform TWAS. Given that the number of genes is significantly lower than the number of common variants, TWAS collapses the burden of multiple hypothesis testing. It also provides the direction of effect which important for understanding how it chould be treated as a potential drug target.

Gene expression for the summary statistics of a trait is imputed by combining variants with eQTL predictive panels from specific tissues. The eQTL predictive panels are constructed from existing datasets of individuals who had been genotyped and also had gene expression measured in various tissues. Each panel is tissue specific. The resulting imputed dataset of genetic variation and imputed gene expression is used to perform a TWAS. The TWAS identifies associations between gene expression and the trait of interest.

The statistical associations are then estimated between predicted gene expression and the trait. Michael et al. 2019 performed a large TWAS for SZ, which was based on RNA-Seq data from the PsychEncode Consortium, 64 genes were found to be significant associated with SZ, including several interesting novel candidates for SZ, *SLC12A5* which encodes a mitochondrial Ca2+ binding aspartate/glutamate carrier protein; *LINC00634*, a downregulated poorly annotated brain-enriched Long intergenic non-coding RNA; *RERE*, a downregulated, mutationally intolerant nuclear receptor coregulator of retinoic acid signaling associated with a rare neurodevelopmental genetic syndrome and downregulated lysine methyltransferases (*SETD6, SETD8*). Among these genes *CLCN3, ZNF804A* and *SNAP91* mentioned earlier as associated with behavioural phenotypes in knockout studies [102, 103].

## Validation and functional analysis

While computational analyses provide important leads on the SNPs most likely to be causal variants within associated loci, functional validation in the laboratory can provide further support of causality. Several techniques now provide an opportunity to model the functional significance of candidate SNPs, both in vitro and in vivo. Together, these experimental approaches can help to provide definitive evidence of causality for SNPs that have been identified as potential causal variants through computational analyses in GWAS. These intervention studies provide empirical support that causal variants effect phenotypes relevant to disease.

## Modelling protein DNA interactions

Recently, it has been shown that regulatory regions are significantly enriched for SZ-associated variants [104, 105], this suggests that genetic variants in non-coding areas confer risk of SZ by disrupting regulatory function. This hypothesis has been supported by accumulating evidence that the majority of risk variants associated with SZ by GWAS affect gene expression rather than protein function or structure. So far there are only a few reports identifying functional variants [106–108]. The non-coding DNA, including promoters and enhancers play a crucial role in gene expression control. Based on previous research, the majority of associations found in GWAS were ascribable to variants located in regulatory elements [24, 48]. An important way to identify functional variants for complex diseases is to investigate whether the risk variants are localised in regulatory elements [109, 110]. The multiple binding sites usually are present in regulatory elements for TFs and gene expression is affected through genetic variations in regulatory elements to directly alter binding affinities of TFs. Several approaches have been developed, including Chromatin immunoprecipitation (ChIP) assays and electrophoretic mobility shift assays (EMSAs) [111], for investigating the binding affinities of noncoding variants with regulatory binding proteins. EMSA has commonly been known as gel shift assays, in which a fluorescent probe, or radiolabeled, DNA oligonucleotide sequence are mixed and incubated with cell lysate of interesting cell types. The mixture is then run with gel electrophoresis to separate unbound DNA from DNA-protein complexes. Moreover, an antibody for a specific protein is also subjected to gel electrophoresis with a mixture of sequence and lysate for supporting the involvement of a given candidate protein in a protein-DNA complex. The anticipation is that the antibody will either hinder the DNA-protein complex or induce a supershift in the complex (resulting in the formation of a triple complex that migrates differently on the gel) [112].

A significant limitation of EMSAs is that these experiments are in vitro and may not accurately reflect conditions in the cell nucleus and what happens to endogenous DNA. To overcome this limitation, ChIP assays can be used for investigation of the interaction between the proteome and the genome by monitoring regulation of transcription through TF–DNA binding interactions or histone modification in the cells. This is followed by isolation of specific antibodies with the candidate protein, retrieval of any DNA sequences cross-linked to the purified protein and performing DNA sequence analysis. For establishing that a DNA variant affects the candidate factor binding, one should demonstrate with ChIP that the factor is preferentially binding to a sequence with 1 allele compared with the alternate allele [113].

## Reporter gene assays

Another method for investigating the relationship between non-coding genetic variation and transcriptional regulatory activity is the reporter assay [114]. In this procedure, regulatory regions surrounding identified candidate variants are engineered into a reported plasmid so that they might regulate a reporter gene, usually a fluorescent or luminescent protein, is transiently expressed in a model organism or cell line. The regulatory activity for each construct can then be measured by comparing their reporter activity. More recently researchers have been able to validate tens of thousands of genetic variants in one experiment in a massive parallel reporter assay (MPRA) which leverages high throughput cloning strategies and RNA-Sequencing [115, 116]. In the context of MPRA, it becomes possible to concurrently assess the functionality of numerous potential regulatory sequences. These sequences, when considered as candidates, are replicated just before the reporter gene. Each sequence is accompanied by a distinct barcode and then inserted into cells. If a candidate regulatory sequence is operational, it prompts the transcription of the linked barcode sequence. This process is evaluated through RNA sequencing and DNA sequencing of the barcode, with adjustments made to account for any imbalanced distributions of plasmid clones or viral inserts. Through the application of this methodology, we gain the capacity to examine a vast array of sequences and their variations to understand their regulatory effects, consequently unraveling the regulatory code and its evolutionary path.

In a recent study, EMSA and dual-luciferase promoter assays were employed to investigate gene-gene interactions (epistasis) and their functional effects. The study focused on a network of interacting dopaminergic polymorphisms that may increase the risk of SZ. EMSA analyses were conducted using rs464049 probes in neuroblastoma cell lines, and specific bandshift patterns were identified, suggesting that sequences flanking rs3756450 could represent a novel promoter domain for dopaminergic genes, such as *SLC6A3* [117].

## Genome editing

Experimental approaches described above have the main limitation, that they do not test the variants function in the native genomic context, which might therefore result in a large proportion of false-negative and false-positive results. Based on these considerations, genome editing might be a more physiologically relevant method for investigating variants functions, harnessing DNA double-strand break (DSB) repair pathways, yielding desired genomic alterations within cells and organisms.

There are two ways of repairing DNA DSBs: non-homologous end joining (NHEJ) or homology-directed repair (HDR) using a donor DNA, the default pathway for repairing DNA DSBs is NHEJ, which typically leads to the formation of deletions and short insertions (indels) at the cleavage site, spanning only a few base pairs. Conversely, HDR is commonly acknowledged as a highly accurate pathway that can utilise a repair template to generate exact genetic alterations. However, partial homology-driven repair events and competing NHEJ repair events often lead to a range of on-target alleles being produced by genome editing experiments. There have been extensive reviews on the DNA repair pathways that are involved in genome editing and the strategies used to favor various outcomes [118].

To date, a number of genome-editing tools have become widely used in recent years, including transcription activator–like effector nucleases (TALENs) [119, 120], zinc-finger nucleases (ZFNs) [119, 121] and the RNA-guided CRISPR-Cas nuclease system [122, 123]. CRISPR-Cas9 (Clustered Regularly Interspaced Short Palindromic Repeats/CRISPR associated protein 9) systems have become particularly popular because of their efficacy and ease of use compared with other tools. CRISPR is a revolutionary methodology that enables the construction of isogenic cell lines with very specific mutations facilitating direct comparison of their effect on gene expression independently of their usual context within their haplotype. Where these experiments are able to establish a role for specific sequence variation in gene dysregulation, these isogenic cell lines can then be further investigated to explore their biological function with isogenic comparison cells. This is a very powerful approach, as cells with causal variants will essentially be compared with isogenic clones without this causal variant. Conversely, specific risk variants can be introduced to a cell line generated from an individual without psychiatric illness, to directly compare the effect of this alteration in their genome.

A study on the spatial organization of chromatin in the fetal human brain found that a region identified in a SZ GWAS interacts with the promoter of FOXG1, which is located 750 kb away from it [124]. A later investigation revealed that the FOXG1-interacting single nucleotide polymorphism (SNP) rs1191551 is in proximity to one of the ATAC-seq peaks detected in the fetal brain [125]. A functional test, specifically a luciferase assay, showed that a genomic fragment containing rs1191551 had enhancer activity. To further confirm the involvement of this region in regulating FOXG1, neural progenitor cells were used to perform CRISPR-Cas9 deletion of the 500 bp region surrounding rs1191551. This deletion resulted in a noticeable reduction in the expression of FOXG1 but did not affect the expression of any neighboring gene.

## Single-cell RNA sequencing

Another powerful approach in the context of post-GWAS analyses is single-cell RNA sequencing (scRNA-seq) [126]. This fancy technique allows us to see gene expression at the individual cell level. Unlike bulk RNA sequencing which averages gene expression across thousands of cells, scRNA-seq shows the cellular heterogeneity within tissues. This allows for the classification of cells into distinct types and states based on their gene expression, facilitating the construction of an empirical taxonomy of cell types within a tissue. This level of detail allows you to classify cells into different types and states based on their gene expression and build an empirical taxonomy of cell types within a tissue.

scRNA-seq is particularly useful in post-GWAS analysis because GWAS identify genomic loci associated with disease but do not tell you which cell types those loci affect. By combining GWAS with scRNA-seq you can map those loci to specific cell types and understand the cellular context of genetic risk factors. This is done by looking at the expression specificity of genes within those loci across different cell types and figure out which cells are most implicated in the disease.

Skene et al used scRNA-seq to map SZ associated genomic loci to specific brain cell types. They found that SZ common-variant genomic results always mapped to pyramidal cells, medium spiny neurons (MSNs) and certain interneurons. This allowed for a more precise understanding of the cellular basis of SZ and showed that these specific neuron types are critical to the genetic risk of the disorder [127].

Building on this approach, Duncan et al applied an independent large-scale integrative analysis combining GWAS with a brain-wide single-nucleus RNA-seq dataset comprising over 3 million human brain nuclei from 105 regions. Their findings confirmed the involvement of similar neuronal populations including somatostatin-expressing interneurons and eccentric medium spiny neurons and further proposed a cell-type-based framework for classifying complex brain disorders [128].

Together, these studies show how scRNA-seq closes the gap between GWAS and functional cellular biology and allows you to identify target cells for further experimental modeling and therapeutic intervention.

## Discussion

While the number of genetic associations with SZ through GWAS has grown exponentially in recent years, finding the actual causal variants is still a big problem. Post-GWAS analysis can help with this by explaining the mechanisms and how these variants contribute to the disease. One of the keys to this is understanding how genetic variation impacts SZ, since many of the significant SNPs identified by GWAS are in non-coding regions so interpretation is harder.

In this case we should also mention emerging areas of research that complement our approach. Deep sequencing, for example, can capture rare variants and copy number variations (CNVs) that may not be picked up by GWAS but have big impact on disease risk. These variants although less confounded by linkage disequilibrium present different challenges for functional annotation. As such these were not covered in this review as they involve mechanisms that are outside the scope of our consideration of common variants and causality.

Also cross disorder mapping and quantitative trait mapping, including cognitive function and symptom profile (positive/negative symptoms, treatment response) provide phenotypic information. These are an important direction for future studies on SZ and may inform functional annotation of associated variants.

It is also important to recognize that schizophrenia is presumed to have a neurodevelopmental etiology, and many risk loci and genes identified through post-GWAS methods show temporal and spatial patterns of expression during prenatal and early brain development [129]. Incorporating this developmental context when interpreting prioritized loci and genes may enhance our understanding of their biological relevance and translational potential.

Advanced computational and experimental methods will be needed to confirm the role of these variants. For example genome editing tools like CRISPR can be used to empirically test the effect of individual genetic changes on gene expression. With further development these can be used to validate suspected causal variants. But integrating findings from deep sequencing, CNV studies and phenotypic mapping will be equally important to get a full picture of SZ.

## References

[1] McGrath J, Saha S, Chant D, Welham J. Schizophrenia: a concise overview of incidence, prevalence, and mortality. Epidemiol Rev. 2008;30:67–76. 18480098 10.1093/epirev/mxn001

[2] Valton V, Romaniuk L, Douglas Steele J, Lawrie S, Seriès P. Comprehensive review: computational modelling of schizophrenia. Neurosci Biobehav Rev. 2017;83:631–46. 28867653 10.1016/j.neubiorev.2017.08.022

[3] Howes OD, Kapur S. The dopamine hypothesis of schizophrenia: version III-the final common pathway. Schizophr Bull. 2009;35:549–62. 19325164 10.1093/schbul/sbp006 PMC2669582

[4] Olney JW, Farber NB. Glutamate receptor dysfunction and schizophrenia. Arch Gen Psychiatry. 1995;52:998–1007. 7492260 10.1001/archpsyc.1995.03950240016004

[5] Delini-Stula A, Berdah-Tordjman D. Benzodiazepines and GABA hypothesis of schizophrenia. J Psychopharmacol. 1995;9:57–63. 22298694 10.1177/026988119500900109

[6] Eggers AE. A serotonin hypothesis of schizophrenia. Med Hypotheses. 2013;80:791–4. 23557849 10.1016/j.mehy.2013.03.013

[7] Avramopoulos D, Pearce BD, McGrath J, Wolyniec P, Wang R, Eckart N, et al. Infection and inflammation in schizophrenia and bipolar disorder: a genome wide study for interactions with genetic variation. PLoS One. 2015;10:e0116696. 25781172 10.1371/journal.pone.0116696 PMC4363491

[8] Brown AS. Prenatal infection as a risk factor for schizophrenia. Schizophr Bull. 2006;32:200–2. 16469941 10.1093/schbul/sbj052 PMC2632220

[9] Insel TR. Rethinking schizophrenia. Nature. 2010;468:187–93. 21068826 10.1038/nature09552

[10] Owen MJ, O’Donovan MC, Thapar A, Craddock N. Neurodevelopmental hypothesis of schizophrenia. Br J Psychiatry. 2011;198:173–5. 21357874 10.1192/bjp.bp.110.084384 PMC3764497

[11] Polderman TJ, Benyamin B, de Leeuw CA, Sullivan PF, van Bochoven A, Visscher PM, et al. Meta-analysis of the heritability of human traits based on fifty years of twin studies. Nat Genet. 2015;47:702–9. 25985137 10.1038/ng.3285

[12] Visscher PM, Goddard ME, From RA. Fisher’s 1918 paper to GWAS a century later. Genetics. 2019;211:1125–30. 30967441 10.1534/genetics.118.301594 PMC6456325

[13] Golan D, Lander ES, Rosset S. Measuring missing heritability: inferring the contribution of common variants. Proc Natl Acad Sci USA. 2014;111:E5272–81. 25422463 10.1073/pnas.1419064111 PMC4267399

[14] Tam V, Patel N, Turcotte M, Bossé Y, Paré G, Meyre D. Benefits and limitations of genome-wide association studies. Nat Rev Genet. 2019;20:467–84. 31068683 10.1038/s41576-019-0127-1

[15] Duan J, Sanders AR, Gejman PV. Genome-wide approaches to schizophrenia. Brain Res Bull. 2010;83:93–102. 20433910 10.1016/j.brainresbull.2010.04.009 PMC2941569

[16] O’donovan MC, Craddock N, Norton N, Williams H, Peirce T, Moskvina V, et al. Identification of loci associated with schizophrenia by genome-wide association and follow-up. Nat Genet. 2008;40:1053–5. 18677311 10.1038/ng.201

[17] Ripke S, Neale BM, Corvin A, Walters JTR, Farh K-H, Holmans PA, et al. Biological insights from 108 schizophrenia-associated genetic loci. Nature. 2014;511:421–7. 25056061 10.1038/nature13595 PMC4112379

[18] Trubetskoy V, Pardiñas AF, Qi T, Panagiotaropoulou G, Awasthi S, Bigdeli TB, et al. Mapping genomic loci implicates genes and synaptic biology in schizophrenia. Nature. 2022;604:502–8. 35396580 10.1038/s41586-022-04434-5 PMC9392466

[19] Kumar V, Wijmenga C, Withoff S. From genome-wide association studies to disease mechanisms: celiac disease as a model for autoimmune diseases. Semin Immunopathol. 2012;34:567–80. 22580835 10.1007/s00281-012-0312-1 PMC3410018

[20] Stefansson H, Ophoff RA, Steinberg S, Andreassen OA, Cichon S, Rujescu D, et al. Common variants conferring risk of schizophrenia. Nature. 2009;460:744–7. 19571808 10.1038/nature08186 PMC3077530

[21] Sullivan PF, Lin D, Tzeng J-Y, van den Oord E, Perkins D, Stroup TS, et al. Genomewide association for schizophrenia in the CATIE study: results of stage 1. Mol Psychiatry. 2008;13:570–84. 18347602 10.1038/mp.2008.25 PMC3910086

[22] Consortium S.. Genome-wide association study identifies five new schizophrenia loci. Nat Genet. 2011;43:969–76. 21926974 10.1038/ng.940 PMC3303194

[23] Ripke S, O’Dushlaine C, Chambert K, Moran JL, Kähler AK, Akterin S, et al. Genome-wide association analysis identifies 13 new risk loci for schizophrenia. Nat Genet. 2013;45:1150. 23974872 10.1038/ng.2742 PMC3827979

[24] Consortium SWGotPG. Biological insights from 108 schizophrenia-associated genetic loci. Nature. 2014;511:421–7. 25056061 10.1038/nature13595 PMC4112379

[25] Pardiñas AF, Holmans P, Pocklington AJ, Escott-Price V, Ripke S, Carrera N, et al. Common schizophrenia alleles are enriched in mutation-intolerant genes and in regions under strong background selection. Nat Genet. 2018;50:381–9. 29483656 10.1038/s41588-018-0059-2 PMC5918692

[26] Wray NR, Yang J, Hayes BJ, Price AL, Goddard ME, Visscher PM. Pitfalls of predicting complex traits from SNPs. Nat Rev Genet. 2013;14:507–15. 23774735 10.1038/nrg3457 PMC4096801

[27] Nyegaard M, Demontis D, Foldager L, Hedemand A, Flint TJ, Sørensen KM, et al. CACNA1C (rs1006737) is associated with schizophrenia. Mol Psychiatry. 2010;15:119–21. 20098439 10.1038/mp.2009.69

[28] Bhat S, Dao DT, Terrillion CE, Arad M, Smith RJ, Soldatov NM, et al. CACNA1C (Cav1. 2) in the pathophysiology of psychiatric disease. Prog Neurobiol. 2012;99:1–14. 22705413 10.1016/j.pneurobio.2012.06.001 PMC3459072

[29] Freedman ML, Monteiro AN, Gayther SA, Coetzee GA, Risch A, Plass C, et al. Principles for the post-GWAS functional characterization of cancer risk loci. Nat Genet. 2011;43:513–8. 21614091 10.1038/ng.840 PMC3325768

[30] Bradshaw NJ, Porteous DJ. DISC1-binding proteins in neural development, signalling and schizophrenia. Neuropharmacology. 2012;62:1230–41. 21195721 10.1016/j.neuropharm.2010.12.027 PMC3275753

[31] Mongan D, Sabherwal S, Susai SR, Föcking M, Cannon M, Cotter DR. Peripheral complement proteins in schizophrenia: a systematic review and meta-analysis of serological studies. Schizophr Res. 2020;222:58–72. 32456884 10.1016/j.schres.2020.05.036 PMC7594643

[32] Chimusa ER, Dalvie S, Dandara C, Wonkam A, Mazandu GK. Post genome-wide association analysis: dissecting computational pathway/network-based approaches. Brief Bioinform. 2018;20:690–700. 10.1093/bib/bby035 PMC6556901 29701762

[33] Ioannidis JP, Trikalinos TA, Khoury MJ. Implications of small effect sizes of individual genetic variants on the design and interpretation of genetic association studies of complex diseases. Am J Epidemiol. 2006;164:609–14. 16893921 10.1093/aje/kwj259

[34] Zeggini E, Ioannidis JP. Meta-analysis in genome-wide association studies. Pharmacogenomics. 2009;10:191–201. 19207020 10.2217/14622416.10.2.191 PMC2695132

[35] Pfeiffer RM, Gail MH, Pee D. On combining data from genome-wide association studies to discover disease-associated SNPs. Stat Sci. 2009;24:547–60.

[36] Pereira TV, Patsopoulos NA, Salanti G, Ioannidis JP. Discovery properties of genome-wide association signals from cumulatively combined data sets. Am J Epidemiol. 2009;170:1197–206. 19808636 10.1093/aje/kwp262 PMC2800267

[37] Ioannidis JP, Patsopoulos NA, Evangelou E. Heterogeneity in meta-analyses of genome-wide association investigations. PLoS One. 2007;2:e841. 17786212 10.1371/journal.pone.0000841 PMC1950790

[38] Faye LL, Machiela MJ, Kraft P, Bull SB, Sun L. Re-ranking sequencing variants in the post-GWAS era for accurate causal variant identification. PLoS Genet. 2013;9:e1003609. 23950724 10.1371/journal.pgen.1003609 PMC3738448

[39] Yang J, Ferreira T, Morris AP, Medland SE, Madden PAF, Heath AC, et al. Conditional and joint multiple-SNP analysis of GWAS summary statistics identifies additional variants influencing complex traits. Nat Genet. 2012;44:369–75. 22426310 10.1038/ng.2213 PMC3593158

[40] Schaid DJ, Chen W, Larson NB. From genome-wide associations to candidate causal variants by statistical fine-mapping. Nat Rev Genet. 2018;19:491–504. 29844615 10.1038/s41576-018-0016-z PMC6050137

[41] Stephens M, Balding DJ. Bayesian statistical methods for genetic association studies. Nat Rev Genet. 2009;10:681–90. 19763151 10.1038/nrg2615

[42] Morris AP. Transethnic meta-analysis of genomewide association studies. Genet Epidemiol. 2011;35:809–22. 22125221 10.1002/gepi.20630 PMC3460225

[43] Kichaev G, Pasaniuc B. Leveraging functional-annotation data in trans-ethnic fine-mapping studies. American J Hum Genet. 2015;97:260–71. 10.1016/j.ajhg.2015.06.007 PMC4573268 26189819

[44] Han B, Eskin E. Random-effects model aimed at discovering associations in meta-analysis of genome-wide association studies. American J Hum Genet. 2011;88:586–98. 10.1016/j.ajhg.2011.04.014 PMC3146723 21565292

[45] Lam M, Chen C-Y, Li Z, Martin AR, Bryois J, Ma X, et al. Comparative genetic architectures of schizophrenia in east asian and european populations. Nat Genet. 2019;51:1670–8. 31740837 10.1038/s41588-019-0512-x PMC6885121

[46] Dunham I, Kundaje A, Aldred SF, Collins PJ, Davis CA, Doyle F, et al. An integrated encyclopedia of DNA elements in the human genome. Nature. 2012;489:57–74. 22955616 10.1038/nature11247 PMC3439153

[47] Bernstein BE, Stamatoyannopoulos JA, Costello JF, Ren B, Milosavljevic A, Meissner A, et al. The NIH roadmap epigenomics mapping consortium. Nat Biotechnol. 2010;28:1045–8. 20944595 10.1038/nbt1010-1045 PMC3607281

[48] Maurano MT, Humbert R, Rynes E, Thurman RE, Haugen E, Wang H, et al. Systematic localization of common disease-associated variation in regulatory DNA. Science. 2012;337:1190–5. 22955828 10.1126/science.1222794 PMC3771521

[49] Wang G-J, Yang P, Xie H-G. Gene variants in noncoding regions and their possible consequences. Pharmacogenomics. 2006;7:203–9. 16515399 10.2217/14622416.7.2.203

[50] Yao L, Tak YG, Berman BP, Farnham PJ. Functional annotation of colon cancer risk SNPs. Nat Commun. 2014;5:1–13. 10.1038/ncomms6114 PMC4200523 25268989

[51] Coetzee SG, Pierce S, Brundin P, Brundin L, Hazelett DJ, Coetzee GA. Enrichment of risk SNPs in regulatory regions implicate diverse tissues in Parkinson’s disease etiology. Sci Rep. 2016;6:1–11. 27461410 10.1038/srep30509 PMC4962314

[52] Pickrell JK. Joint analysis of functional genomic data and genome-wide association studies of 18 human traits. American J Hum Genet. 2014;94:559–73. 10.1016/j.ajhg.2014.03.004 PMC3980523 24702953

[53] Kichaev G, Yang W-Y, Lindstrom S, Hormozdiari F, Eskin E, Price AL, et al. Integrating functional data to prioritize causal variants in statistical fine-mapping studies. PLoS Genet. 2014;10:e1004722. 25357204 10.1371/journal.pgen.1004722 PMC4214605

[54] Trynka G, Sandor C, Han B, Xu H, Stranger BE, Liu XS, et al. Chromatin marks identify critical cell types for fine mapping complex trait variants. Nat Genet. 2013;45:124–30. 23263488 10.1038/ng.2504 PMC3826950

[55] Chung D, Yang C, Li C, Gelernter J, Zhao H. GPA: a statistical approach to prioritizing GWAS results by integrating pleiotropy and annotation. PLoS Genet. 2014;10:e1004787. 25393678 10.1371/journal.pgen.1004787 PMC4230845

[56] Niu H-M, Yang P, Chen H-H, Hao R-H, Dong S-S, Yao S, et al. Comprehensive functional annotation of susceptibility SNPs prioritized 10 genes for schizophrenia. Transl Psychiatry. 2019;9:1–12. 30705251 10.1038/s41398-019-0398-5 PMC6355777

[57] Hindorff LA, Sethupathy P, Junkins HA, Ramos EM, Mehta JP, Collins FS, et al. Potential etiologic and functional implications of genome-wide association loci for human diseases and traits. Proceedings Natl Acad Sci. 2009;106:9362–7. 10.1073/pnas.0903103106 PMC2687147 19474294

[58] Califano A, Butte AJ, Friend S, Ideker T, Schadt E. Leveraging models of cell regulation and GWAS data in integrative network-based association studies. Nat Genet. 2012;44:841–7. 22836096 10.1038/ng.2355 PMC3593099

[59] Hall J, Trent S, Thomas KL, O’Donovan MC, Owen MJ. Genetic risk for schizophrenia: convergence on synaptic pathways involved in plasticity. Biol Psychiatry. 2015;77:52–8. 25152434 10.1016/j.biopsych.2014.07.011

[60] Chen X, Wang L, Hu B, Guo M, Barnard J, Zhu X. Pathway-based analysis for genome-wide association studies using supervised principal components. Genet Epidemiol. 2010;34:716–24. 20842628 10.1002/gepi.20532 PMC3480088

[61] Guo Y-F, Li J, Chen Y, Zhang L-S, Deng H-W. A new permutation strategy of pathway-based approach for genome-wide association study. BMC Bioinforma. 2009;10:429. 10.1186/1471-2105-10-429 PMC2809078 20021635

[62] de Leeuw CA, Neale BM, Heskes T, Posthuma D. The statistical properties of gene-set analysis. Nat Rev Genet. 2016;17:353–64. 27070863 10.1038/nrg.2016.29

[63] Nurnberger JI Jr, Koller DL, Jung J, Edenberg HJ, Foroud T, Guella I, et al. Identification of pathways for bipolar disorder: a meta-analysis. JAMA Psychiatry. 2014;71:657–64. 24718920 10.1001/jamapsychiatry.2014.176 PMC4523227

[64] Askland K, Read C, Moore J. Pathways-based analyses of whole-genome association study data in bipolar disorder reveal genes mediating ion channel activity and synaptic neurotransmission. Hum Genet. 2009;125:63–79. 19052778 10.1007/s00439-008-0600-y

[65] Menashe I, Maeder D, Garcia-Closas M, Figueroa JD, Bhattacharjee S, Rotunno M, et al. Pathway analysis of breast cancer genome-wide association study highlights three pathways and one canonical signaling cascade. Cancer Res. 2010;70:4453–9. 20460509 10.1158/0008-5472.CAN-09-4502 PMC2907250

[66] Manolio TA. Bringing genome-wide association findings into clinical use. Nat Rev Genet. 2013;14:549–58. 23835440 10.1038/nrg3523

[67] Qian DC, Byun J, Han Y, Greene CS, Field JK, Hung RJ, et al. Identification of shared and unique susceptibility pathways among cancers of the lung, breast, and prostate from genome-wide association studies and tissue-specific protein interactions. Hum Mol Genet. 2015;24:7406–20. 26483192 10.1093/hmg/ddv440 PMC4664175

[68] Network and Pathway Analysis Subgroup of Psychiatric Genomics Consortium. Psychiatric genome-wide association study analyses implicate neuronal, immune and histone pathways. Nat Neurosci. 2015;18:199–209. 25599223 10.1038/nn.3922 PMC4378867

[69] Hertzberg L, Katsel P, Roussos P, Haroutunian V, Domany E. Integration of gene expression and GWAS results supports involvement of calcium signaling in Schizophrenia. Schizophr Res. 2015;164:92–9. 25702973 10.1016/j.schres.2015.02.001

[70] Fabregat A, Jupe S, Matthews L, Sidiropoulos K, Gillespie M, Garapati P, et al. The reactome pathway knowledgebase. Nucleic Acids Res. 2018;46:D649–D55. 29145629 10.1093/nar/gkx1132 PMC5753187

[71] Ashburner M, Ball CA, Blake JA, Botstein D, Butler H, Cherry JM, et al. Gene ontology: tool for the unification of biology. Nat Genet. 2000;25:25–9. 10802651 10.1038/75556 PMC3037419

[72] Kanehisa M, Goto S, Sato Y, Furumichi M, Tanabe M. KEGG for integration and interpretation of large-scale molecular data sets. Nucleic Acids Res. 2012;40:D109–D14. 22080510 10.1093/nar/gkr988 PMC3245020

[73] Darnell JC, Van Driesche SJ, Zhang C, Hung KYS, Mele A, Fraser CE, et al. FMRP stalls ribosomal translocation on mRNAs linked to synaptic function and autism. Cell. 2011;146:247–61. 21784246 10.1016/j.cell.2011.06.013 PMC3232425

[74] Müller CS, Haupt A, Bildl W, Schindler J, Knaus H-G, Meissner M, et al. Quantitative proteomics of the Cav2 channel nano-environments in the mammalian brain. Proceedings Natl Acad Sci. 2010;107:14950–7. 10.1073/pnas.1005940107 PMC2930569 20668236

[75] Bécamel C, Alonso G, Galéotti N, Demey E, Jouin P, Ullmer C, et al. Synaptic multiprotein complexes associated with 5-HT2C receptors: a proteomic approach. EMBO J. 2002;21:2332–42. 12006486 10.1093/emboj/21.10.2332 PMC126011

[76] Purcell SM, Moran JL, Fromer M, Ruderfer D, Solovieff N, Roussos P, et al. A polygenic burden of rare disruptive mutations in schizophrenia. Nature. 2014;506:185–90. 24463508 10.1038/nature12975 PMC4136494

[77] Schijven D, Kofink D, Tragante V, Verkerke M, Pulit SL, Kahn RS, et al. Comprehensive pathway analyses of schizophrenia risk loci point to dysfunctional postsynaptic signaling. Schizophr Res. 2018;199:195–202. 29653892 10.1016/j.schres.2018.03.032

[78] Lee YH, Kim J-H, Song GG. Pathway analysis of a genome-wide association study in schizophrenia. Gene. 2013;525:107–15. 23644028 10.1016/j.gene.2013.04.014

[79] O’Dushlaine C, Kenny E, Heron E, Donohoe G, Gill M, Morris D, et al. Molecular pathways involved in neuronal cell adhesion and membrane scaffolding contribute to schizophrenia and bipolar disorder susceptibility. Mol Psychiatry. 2011;16:286–92. 20157312 10.1038/mp.2010.7

[80] Meguro-Horike M, Yasui DH, Powell W, Schroeder DI, Oshimura M, LaSalle JM, et al. Neuron-specific impairment of inter-chromosomal pairing and transcription in a novel model of human 15q-duplication syndrome. Hum Mol Genet. 2011;20:3798–810. 21725066 10.1093/hmg/ddr298 PMC3168289

[81] Sopher BL, Ladd PD, Pineda VV, Libby RT, Sunkin SM, Hurley JB, et al. CTCF regulates ataxin-7 expression through promotion of a convergently transcribed, antisense noncoding RNA. Neuron. 2011;70:1071–84. 21689595 10.1016/j.neuron.2011.05.027 PMC3139428

[82] De Souza RA, Islam SA, McEwen LM, Mathelier A, Hill A, Mah SM, et al. DNA methylation profiling in human Huntington’s disease brain. Hum Mol Genet. 2016;25:2013–30. 26953320 10.1093/hmg/ddw076

[83] Ripke S, O’Dushlaine C, Chambert K, Moran JL, Kähler AK, Akterin S, et al. Genome-wide association analysis identifies 14 new risk loci for schizophrenia. Nat Genet. 2013;45:1150–9. 23974872 10.1038/ng.2742 PMC3827979

[84] Roussos P, Mitchell Amanda C, Voloudakis G, Fullard John F, Pothula Venu M, Tsang J, et al. A role for noncoding variation in schizophrenia. Cell Rep. 2014;9:1417–29. 25453756 10.1016/j.celrep.2014.10.015 PMC4255904

[85] Civelek M, Lusis AJ. Systems genetics approaches to understand complex traits. Nat Rev Genet. 2014;15:34–48. 24296534 10.1038/nrg3575 PMC3934510

[86] Garrido-Cardenas JA, Mesa-Valle C, Manzano-Agugliaro F. Trends in plant research using molecular markers. Planta. 2018;247:543–57. 29243155 10.1007/s00425-017-2829-y

[87] Ye Y, Zhang Z, Liu Y, Diao L, Han L. A multi-omics perspective of quantitative trait loci in precision medicine. Trends Genet. 2020;36:318–36. 32294413 10.1016/j.tig.2020.01.009

[88] Vandiedonck C. Genetic association of molecular traits: A help to identify causative variants in complex diseases. Clin Genet. 2018;93:520–32. 29194587 10.1111/cge.13187

[89] Li MJ, Yan B, Sham PC, Wang J. Exploring the function of genetic variants in the non-coding genomic regions: approaches for identifying human regulatory variants affecting gene expression. Brief Bioinform. 2014;16:393–412. 24916300 10.1093/bib/bbu018

[90] Cano-Gamez E, Trynka G. From GWAS to function: using functional genomics to identify the mechanisms underlying complex diseases. Front Genet. 2020;11:424. 32477401 10.3389/fgene.2020.00424 PMC7237642

[91] Lonsdale J, Thomas J, Salvatore M, Phillips R, Lo E, Shad S, et al. The genotype-tissue expression (GTEx) project. Nat Genet. 2013;45:580–5. 23715323 10.1038/ng.2653 PMC4010069

[92] Fromer M, Roussos P, Sieberts SK, Johnson JS, Kavanagh DH, Perumal TM, et al. Gene expression elucidates functional impact of polygenic risk for schizophrenia. Nat Neurosci. 2016;19:1442–53. 27668389 10.1038/nn.4399 PMC5083142

[93] Zhu Z, Zhang F, Hu H, Bakshi A, Robinson MR, Powell JE, et al. Integration of summary data from GWAS and eQTL studies predicts complex trait gene targets. Nat Genet. 2016;48:481–7. 27019110 10.1038/ng.3538

[94] Nicolae DL, Gamazon E, Zhang W, Duan S, Dolan ME, Cox NJ. Trait-associated SNPs are more likely to be eQTLs: annotation to enhance discovery from GWAS. PLoS Genet. 2010;6:e1000888. 20369019 10.1371/journal.pgen.1000888 PMC2848547

[95] Giambartolomei C, Vukcevic D, Schadt EE, Franke L, Hingorani AD, Wallace C, et al. Bayesian test for colocalisation between pairs of genetic association studies using summary statistics. PLoS Genet. 2014;10:e1004383. 24830394 10.1371/journal.pgen.1004383 PMC4022491

[96] Mao Y, Ge X, Frank CL, Madison JM, Koehler AN, Doud MK, et al. Disrupted in schizophrenia 1 regulates neuronal progenitor proliferation via modulation of GSK3β/β-catenin signaling. Cell. 2009;136:1017–31. 19303846 10.1016/j.cell.2008.12.044 PMC2704382

[97] Hashimoto-Torii K, Torii M, Sarkisian MR, Bartley CM, Shen J, Radtke F, et al. Interaction between reelin and notch signaling regulates neuronal migration in the cerebral cortex. Neuron. 2008;60:273–84. 18957219 10.1016/j.neuron.2008.09.026 PMC2913541

[98] Yang C-P, Li X, Wu Y, Shen Q, Zeng Y, Xiong Q, et al. Comprehensive integrative analyses identify GLT8D1 and CSNK2B as schizophrenia risk genes. Nat Commun. 2018;9:838. 29483533 10.1038/s41467-018-03247-3 PMC5826945

[99] Ma C, Gu C, Huo Y, Li X, Luo X-J. The integrated landscape of causal genes and pathways in schizophrenia. Transl Psychiatry. 2018;8:67. 29540662 10.1038/s41398-018-0114-x PMC5851982

[100] Gusev A, Ko A, Shi H, Bhatia G, Chung W, Penninx BW, et al. Integrative approaches for large-scale transcriptome-wide association studies. Nat Genet. 2016;48:245–52. 26854917 10.1038/ng.3506 PMC4767558

[101] Gamazon ER, Wheeler HE, Shah KP, Mozaffari SV, Aquino-Michaels K, Carroll RJ, et al. A gene-based association method for mapping traits using reference transcriptome data. Nat Genet. 2015;47:1091. 26258848 10.1038/ng.3367 PMC4552594

[102] Gandal MJ, Zhang P, Hadjimichael E, Walker RL, Chen C, Liu S, et al. Transcriptome-wide isoform-level dysregulation in ASD, schizophrenia, and bipolar disorder. Science. 2018;362:eaat8127. 30545856 10.1126/science.aat8127 PMC6443102

[103] Thyme SB, Pieper LM, Li EH, Pandey S, Wang Y, Morris NS, et al. Phenotypic landscape of schizophrenia-associated genes defines candidates and their shared functions. Cell. 2019;177:478–91. e20. 30929901 10.1016/j.cell.2019.01.048 PMC6494450

[104] Liu M, Jiang Y, Wedow R, Li Y, Brazel DM, Chen F, et al. Association studies of up to 1.2 million individuals yield new insights into the genetic etiology of tobacco and alcohol use. Nat Genet. 2019;51:237–44. 30643251 10.1038/s41588-018-0307-5 PMC6358542

[105] Bacanu S, Chen J, Sun J, Richardson K, Lai C, Zhao Z, et al. Functional SNPs are enriched for schizophrenia association signals. Mol Psychiatry. 2014;19:276–7. 23546170 10.1038/mp.2013.33 PMC7954531

[106] Luo X-J, Mattheisen M, Li M, Huang L, Rietschel M, Børglum AD, et al. Systematic integration of brain eQTL and GWAS identifies ZNF323 as a novel schizophrenia risk gene and suggests recent positive selection based on compensatory advantage on pulmonary function. Schizophr Bull. 2015;41:1294–308. 25759474 10.1093/schbul/sbv017 PMC4601704

[107] Li M, Jaffe AE, Straub RE, Tao R, Shin JH, Wang Y, et al. A human-specific AS3MT isoform and BORCS7 are molecular risk factors in the 10q24. 32 schizophrenia-associated locus. Nat Med. 2016;22:649–56. 27158905 10.1038/nm.4096

[108] Sekar A, Bialas AR, De Rivera H, Davis A, Hammond TR, Kamitaki N, et al. Schizophrenia risk from complex variation of complement component 4. Nature. 2016;530:177–83. 26814963 10.1038/nature16549 PMC4752392

[109] Huang Q, Whitington T, Gao P, Lindberg JF, Yang Y, Sun J, et al. A prostate cancer susceptibility allele at 6q22 increases RFX6 expression by modulating HOXB13 chromatin binding. Nat Genet. 2014;46:126–35. 24390282 10.1038/ng.2862

[110] Bond GL, Hu W, Bond EE, Robins H, Lutzker SG, Arva NC, et al. A single nucleotide polymorphism in the MDM2 promoter attenuates the p53 tumor suppressor pathway and accelerates tumor formation in humans. Cell. 2004;119:591–602. 15550242 10.1016/j.cell.2004.11.022

[111] Hellman LM, Fried MG. Electrophoretic mobility shift assay (EMSA) for detecting protein–nucleic acid interactions. Nat Protoc. 2007;2:1849–61. 17703195 10.1038/nprot.2007.249 PMC2757439

[112] Dey B, Thukral S, Krishnan S, Chakrobarty M, Gupta S, Manghani C, et al. DNA–protein interactions: methods for detection and analysis. Mol Cell Biochem. 2012;365:279–99. 22399265 10.1007/s11010-012-1269-z

[113] Mundade R, Ozer HG, Wei H, Prabhu L, Lu T. Role of ChIP-seq in the discovery of transcription factor binding sites, differential gene regulation mechanism, epigenetic marks and beyond. Cell Cycle. 2014;13:2847–52. 25486472 10.4161/15384101.2014.949201 PMC4614920

[114] Trauernicht M, Martinez-Ara M, van Steensel B. Deciphering Gene Regulation Using Massively Parallel Reporter Assays. Trends Biochem Sci. 2020;45:90–1. 31727407 10.1016/j.tibs.2019.10.006

[115] Melnikov A, Zhang X, Rogov P, Wang L, Mikkelsen TS. Massively parallel reporter assays in cultured mammalian cells. J Vis Exp. 2014;17:51719. 10.3791/51719 PMC4364389 25177895

[116] Kreimer A, Zeng H, Edwards MD, Guo Y, Tian K, Shin S, et al. Predicting gene expression in massively parallel reporter assays: a comparative study. Hum Mutat. 2017;38:1240–50. 28220625 10.1002/humu.23197 PMC5560998

[117] Talkowski ME, Kirov G, Bamne M, Georgieva L, Torres G, Mansour H, et al. A network of dopaminergic gene variations implicated as risk factors for schizophrenia. Hum Mol Genet. 2008;17:747–58. 18045777 10.1093/hmg/ddm347 PMC3777405

[118] Rao S, Yao Y, Bauer DE. Editing GWAS: experimental approaches to dissect and exploit disease-associated genetic variation. Genome Med. 2021;13:41. 33691767 10.1186/s13073-021-00857-3 PMC7948363

[119] Wood AJ, Lo T-W, Zeitler B, Pickle CS, Ralston EJ, Lee AH, et al. Targeted genome editing across species using ZFNs and TALENs. Science. 2011;333:307. 21700836 10.1126/science.1207773 PMC3489282

[120] Christian M, Cermak T, Doyle EL, Schmidt C, Zhang F, Hummel A, et al. Targeting DNA double-strand breaks with TAL effector nucleases. Genetics. 2010;186:757–61. 20660643 10.1534/genetics.110.120717 PMC2942870

[121] Miller JC, Holmes MC, Wang J, Guschin DY, Lee Y-L, Rupniewski I, et al. An improved zinc-finger nuclease architecture for highly specific genome editing. Nat Biotechnol. 2007;25:778–85. 17603475 10.1038/nbt1319

[122] Cong L, Ran FA, Cox D, Lin S, Barretto R, Habib N, et al. Multiplex genome engineering using CRISPR/Cas systems. Science. 2013;339:819–23. 23287718 10.1126/science.1231143 PMC3795411

[123] Makarova KS, Haft DH, Barrangou R, Brouns SJ, Charpentier E, Horvath P, et al. Evolution and classification of the CRISPR–Cas systems. Nat Rev Microbiol. 2011;9:467–77. 21552286 10.1038/nrmicro2577 PMC3380444

[124] Won H, de La Torre-Ubieta L, Stein JL, Parikshak NN, Huang J, Opland CK, et al. Chromosome conformation elucidates regulatory relationships in developing human brain. Nature. 2016;538:523–7. 27760116 10.1038/nature19847 PMC5358922

[125] de la Torre-Ubieta L, Stein JL, Won H, Opland CK, Liang D, Lu D, et al. The dynamic landscape of open chromatin during human cortical neurogenesis. Cell. 2018;172:289–304. e18. 29307494 10.1016/j.cell.2017.12.014 PMC5924568

[126] Kolodziejczyk AA, Kim JK, Svensson V, Marioni JC, Teichmann SA. The technology and biology of single-cell RNA sequencing. Mol Cell. 2015;58:610–20. 26000846 10.1016/j.molcel.2015.04.005

[127] Skene NG, Bryois J, Bakken TE, Breen G, Crowley JJ, Gaspar HA, et al. Genetic identification of brain cell types underlying schizophrenia. Nat Genet. 2018;50:825–33. 29785013 10.1038/s41588-018-0129-5 PMC6477180

[128] Duncan LE, Li T, Salem M, Li W, Mortazavi L, Senturk H, et al. Mapping the cellular etiology of schizophrenia and complex brain phenotypes. Nat Neurosci. 2025;28:248–58. 39833308 10.1038/s41593-024-01834-w PMC11802450

[129] Birnbaum R, Weinberger DR. The genesis of schizophrenia: an origin story. American J Psychiatry. 2024;181:482–92. 10.1176/appi.ajp.20240305 38822584

[130] Benner C, Spencer CC, Havulinna AS, Salomaa V, Ripatti S, Pirinen M. FINEMAP: efficient variable selection using summary data from genome-wide association studies. Bioinformatics. 2016;32:1493–501. 26773131 10.1093/bioinformatics/btw018 PMC4866522

[131] He X, Fuller CK, Song Y, Meng Q, Zhang B, Yang X, et al. Sherlock: detecting gene-disease associations by matching patterns of expression QTL and GWAS. Am J Hum Genet. 2013;92:667–80. 23643380 10.1016/j.ajhg.2013.03.022 PMC3644637

[132] Hormozdiari F, Kostem E, Kang EY, Pasaniuc B, Eskin E. Identifying causal variants at loci with multiple signals of association. Genetics. 2014;198:497–508. 25104515 10.1534/genetics.114.167908 PMC4196608

[133] de Leeuw CA, Mooij JM, Heskes T, Posthuma D. MAGMA: generalized gene-set analysis of GWAS data. PLoS Comput Biol. 2015;11:e1004219. 25885710 10.1371/journal.pcbi.1004219 PMC4401657

[134] Lee PH, O’Dushlaine C, Thomas B, Purcell SM. INRICH: interval-based enrichment analysis for genome-wide association studies. Bioinformatics. 2012;28:1797–9. 22513993 10.1093/bioinformatics/bts191 PMC3381960

[135] Segrè AV, Consortium D, Investigators M, Groop L, Mootha VK, Daly MJ, et al. Common inherited variation in mitochondrial genes is not enriched for associations with type 2 diabetes or related glycemic traits. PLoS Genet. 2010;6:e1001058. 20714348 10.1371/journal.pgen.1001058 PMC2920848

[136] Lamparter D, Marbach D, Rueedi R, Kutalik Z, Bergmann S. Fast and rigorous computation of gene and pathway scores from SNP-based summary statistics. PLoS Comput Biol. 2016;12:e1004714. 26808494 10.1371/journal.pcbi.1004714 PMC4726509

[137] Nam D, Kim J, Kim S-Y, Kim S. GSA-SNP: a general approach for gene set analysis of polymorphisms. Nucleic Acids Res. 2010;38:W749–W54. 20501604 10.1093/nar/gkq428 PMC2896081

[138] Liu JZ, Mcrae AF, Nyholt DR, Medland SE, Wray NR, Brown KM, et al. A versatile gene-based test for genome-wide association studies. American J Hum Genet. 2010;87:139–45. 10.1016/j.ajhg.2010.06.009 PMC2896770 20598278

[139] Li MX, Gui HS, Kwan JS, Sham PC. GATES: a rapid and powerful gene-based association test using extended simes procedure. Am J Hum Genet. 2011;88:283–93. 21397060 10.1016/j.ajhg.2011.01.019 PMC3059433

